# Application of distraction osteogenesis in managing bone cysts

**DOI:** 10.1007/s10195-013-0272-9

**Published:** 2013-10-16

**Authors:** Vagif Verdiyev, Farhad Verdiyev

**Affiliations:** Department of Bone Pathology, Scientific-research Institute of Traumatology and Orthopaedics, 32 Abbas Sahhat str., Baku, Azerbaijan

**Keywords:** Bone cysts, Distraction osteogenesis, External fixator

## Abstract

**Background:**

Despite the great amount of research concerning bone cysts, there is still no commonly accepted method of treatment. The aim of this study was to evaluate the effectiveness managing bone cyst with hybrid external fixator by distraction osteogenesis.

**Materials and methods:**

Between 1982 and 2009, 25 patients with unicameral (20 patients) and aneurismal (five patients) bone cysts were treated using this method. Eighteen patients had a history of pathological fracture at the same location. Cysts were located in the humerus, femur, tibia, and radius. Median follow-up was 48 (range 31–91) months. Results were evaluated on plain radiographs according to the classification system of Capanna et al. Functional assessment was done using the modified system recommended by Enneking et al.

**Results:**

In our study group of 25 bone cysts, 15 were classified as completely healed and nine as healed with residual radiolucency. Recurrence was observed in one patient. Absence of response to treatment was not observed. All patients had excellent functional outcomes, except one with recurrence who was rated poor.

**Conclusions:**

As bone cysts are found in long bones in 90–95 % of patients, and taking into account our achieved positive results in almost all patients, we recommend this method of distraction osteogenesis as a treatment option. It is an effective, economical method of treatment, which eliminates deformity and restores bone length, especially in patients with pathologic fractures.

## Introduction

Bone cysts usually present in the pediatric population, and despite its benign nature, more than half of the cases present with pathological fracture [1] and pain, which negatively affects life style, as well as causing continuous trouble for participating in sports, etc. They are one of the most common benign bone lesions in childhood, and in this respect, the problem has important social and health-related issues. Given the varied theories about their occurrence [2–5], bone cysts in the historical frame have dictated various approaches in treatment, from extremely drastic segmental resections [6], intramedullary nailing [7], curettage with grafting or cementing [8], continuous decompression with cannulated screws [9], steroid [10–12] or autogenous bone marrow injection [13, 14], and waiting without intervention. Retrospective analyses of these methods of treatment demonstrate no requirement for drastic approaches, and its optimization requires neither resection nor curettage.

Thus, in an attempt to improve treatment results and decrease the time needed for full recovery, our approach was to destroy the closed cystic cavity and decompress and fill it with distractive regenerative bone tissue, simultaneously eliminating limb-length discrepancy and deformities. The aim of this study was to retrospectively evaluate the effectiveness of treatment for bone cysts using the hybrid external fixator method of distraction osteogenesis.

## Materials and methods

**Fig. 1:**
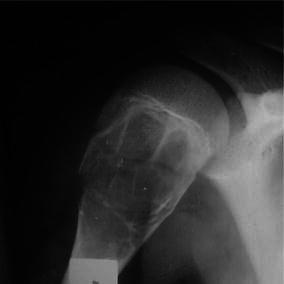
X-ray of active unicameral bone cyst (UBC) with pathological fracture of right humerus in a 14-year-old boy

Between 1982 and 2009, 25 patients with bone cysts were treated in our hospital with distraction osteogenesis. All patients gave informed consent prior to being included in this retrospective study, which was authorized by the local ethical committee and performed in accordance with the Ethical Standards of the 1964 Declaration of Helsinki as revised in 2000. Mean patient age was 12 (7–18) years. There were 22 boys and three girls. Twenty patients had cysts located in the humerus, three in the femur, one in the distal radius and one in the tibia. Cysts were diagnosed radiologically. Unicameral bone cyst (UBC) was diagnosed in 20 and aneurismal bone cyst (ABC) in five patients. All cases were treated with open and half-open methods, and when the diagnosis was in doubt (22 of 25 patients), radiological diagnosis was confirmed histologically. Eighteen patients had a history of pathological fracture at the same location. Craniocaudal length of the cyst in the axis of the bone was 4–14 (mean 8) cm. Ten patients had active cysts that were in direct contact with the adjacent growth plate (Fig. 1); 15 had inactive cysts that were found at a distance from the growth plate (Table 1). Indications for surgery were cysts with large cavities, pathological fractures, growth arrest with deformities, length discrepancy of the affected limb, and absence of allograft.

**Table 1:** Data of patients, with details of cysts and treatment

Patient no.	Sex	Age (years)	Localization	Cyst type	Activity	Pathologic fracture	Craniocaudal length of the cyst (cm)	Method	Time of frame removal (months)	Function	Follow-up (months)
1	M	10	Radius	ABC	Active	No	4	Open	4	Excellent	91
2	M	8	Femur	UBC	Latent	Yes	5	Closed	4	Excellent	89
3	M	14	Humerus	UBC	Active	Yes	7	Open	4	Excellent	89
4	F	8	Humerus	UBC	Latent	Yes	7	Closed	4	Excellent	87
5	M	9	Humerus	UBC	Latent	Yes	7.5	Half open	4	Excellent	84
6	M	9	Humerus	UBC	Latent	Yes	9	Open	4	Excellent	82
7	M	11	Humerus	UBC	Latent	No	14	Open	6	Excellent	75
8	M	11	Humerus	UBC	Latent	Yes	6.5	Half open	4	Excellent	74
9	M	18	Humerus	UBC	Latent	Yes	7	Half open	4	Excellent	74
10	M	18	Humerus	UBC	Active	Yes	10	Open	6	Excellent	73
11	M	15	Humerus	UBC	Latent	No	11.5	Half open	4	Poor	71
12	M	16	Humerus	ABC	Active	No	12	Open	6	Excellent	68
13	M	14	Humerus	UBC	Active	Yes	8	Open	4	Excellent	48
14	F	12	Humerus	ABC	Latent	Yes	11	Half open	6	Excellent	48
15	M	10	Humerus	ABC	Latent	Yes	5	Half open	4	Excellent	46
16	M	7	Humerus	UBC	Latent	Yes	7.5	Open	4	Excellent	45
17	M	17	Humerus	UBC	Active	Yes	7	Open	4	Excellent	44
18	M	11	Humerus	UBC	Active	No	9	Half open	4	Excellent	43
19	F	11	Humerus	ABC	Latent	No	7.5	Half open	4	Excellent	37
20	M	20	Humerus	UBC	Latent	Yes	5	Open	4	Excellent	36
21	M	12	Femur	UBC	Active	Yes	7	Half open	4	Excellent	34
22	M	13	Humerus	ABC	Latent	Yes	9	Open	5	Excellent	34
23	M	14	Femur	UBC	Latent	Yes	9	Closed	5	Excellent	32
24	M	8	Tibia	UBC	Active	No	6	Open	4	Excellent	32
25	M	11	Humerus	UBC	Active	Yes	10	Open	6	Excellent	31

*ABC* aneurismal bone cyst,*UBC* unicameral bone cyst

**Fig. 2:**
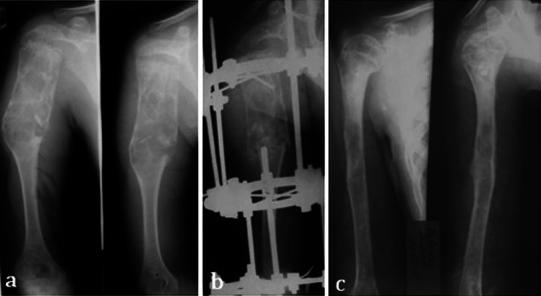
**a** X-ray of aneurysmal bone cyst (ABC) of right humerus in a 13-year-old boy. **b** Application of external fixator and open osteotomy and coaptation.**c** Capanna grade 1, completely healed, after 15 months

All procedures were performed under general anesthesia, with the use of image intensifier or X-ray to localize the cyst and control the position of wires. External fixator wires were inserted proximally and distally and in some cases (six cases) through cystic cavity (for continuous decompression). The external fixator was assembled according to angulatory, translational, and length deformities. Of the 25 patients, we inserted the distal end of the bone to the proximal end by a closed procedure in three patients, a half-open procedure in nine, and an open procedure in remaining 13. We chose the closed method in patients with pathological fracture with a cyst length <7 cm and radiologically different diameters of proximal and distal fracture ends. The half-open method was used in patients with pathological fracture when fractured ends could not be embedded into each other and in patients with thinned cyst wall but without fracture. We performed osteotomy through a 0.5- to 1-cm incision at the distal end of the cyst that had the smaller diameter. The open method was applied in patients with long cysts (generally >7 cm) with thick walls and septations, with or without pathological fracture (Fig. 2). In some of these cases, we performed iatrogenic circular polyfragmented fracture by applying rounded compression with bone forceps, and we used these fragments as local autografts. Curettage was done in patients treated with the open method and was essentially required in patients with cyst septations to create one cystic cavity because isolated cavities prevent proper healing. Relative indication for curettage was an ABC with a soft-tissue component, which was determined by magnetic resonance imaging (MRI) examination. We avoided curettage in patients with active cysts in direct contact with the physis to avoid its damage and consequential deformities and shortenings.

**Fig. 3:**
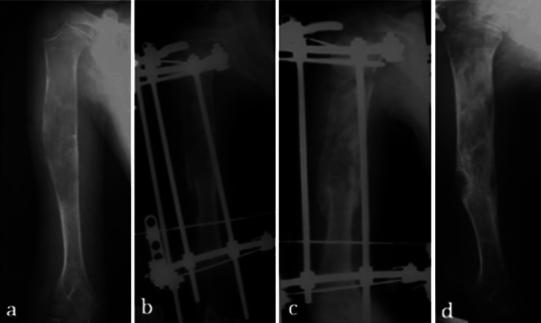
**a** X-ray of unicameral bone cyst (UBC) of right humerus in an 11-year-old boy. **b** Application of external fixator and first-level osteotomy with coaptation. **c** Three months after filling the distal part of the cystic cavity, we performed a second proximal-level osteotomy and distraction. **d** Capanna grade 2 healing with residual cavity

The amount of shortening depends on the length of the cyst, age of the patient, and presence of limb-length discrepancy. It is preferable to compress the length of the cyst by 1 cm to 50 %, not exceeding 3 cm in order to avoid neurovascular injury. Additional compression with neurovascular status monitoring is possible after operation. Prophylactic hypercorrection was done in patients with active cysts and damaged growth plate in which we anticipated shortening and deformities. In one patient with a cyst length in the bone axis of 14 cm (Fig. 3), we performed a two-stage monolocal compression distraction osteogenesis, first in the distal and then in the proximal part of the cyst, given the poor infilling of distal and proximal areas of the cystic cavity.

In two patients, to enforce and strengthen distraction callus, we intraoperatively added hydroxyapatite ceramic in the cystic cavity, and two other patients postoperatively received bisphosphonate therapy; however, due to their small number, those patients were not included in the analysis.

After 7 days postoperatively, we began distraction with 0.25 mm/three times a day. After limb lengthening and deformity correction, we stopped distraction and fixed the apparatus. After radiological consolidation of newly formed bone was achieved, we removed the frame and the patient began physical therapy. No additional cast or bracing was used.

For radiological evaluation, we used the classification system of Cappana et al. [11]. This four-part system is based on response to treatment: (1) healed—cyst completely healed, filled with bone, and thickened cortical margins; (2) healed with residual—cyst consolidated with thickened cortex but persistent areas of osteolysis and cortical thinning; (3) recurrence—cyst showed initial consolidation following treatment but subsequently showed zones of osteolysis and cortical thinning; (4) no response—no evidence of cyst healing. For functional assessment we used the modified system of functional evaluation after surgical management of musculoskeletal tumors recommended by Enneking et al. [15]. This system rating takes into account active range of movement (ROM), pain, stability, deformity, strength, functional activity, and emotional acceptance. For statistical analyses, Kruskal–Wallis test was used to compare means among three groups; Chi-square test was used to compare categorical variables.

## Results

In our study population of 25 bone cysts, 15 (60 %) were classified as completely healed and nine (36 %) as healed with residual radiolucency. Recurrence was observed in one (4 %) patient. Absence of response to treatment was not observed. Median follow-up was 48 (range 31–91) months. In 18 patients, cyst healing and frame removal was within 4 months; in seven patients, this period was 6 months (Table 1). One recurrence was seen 8 months into the postoperative period. The effect of various variables on Capanna outcome is presented in Table 2. Patient age did not influence treatment outcome. Eight patients were ≤10 years, and cysts healed in all cases; 17 patients were >10 years, recurrence occurred in one patient. Of the ten patients with active cysts, only one experienced recurrence; the 15 patients with latent cysts were healed. Patients with pathological fracture had better outcomes than patients without it. No statistically significant differences in healing time and outcome were noted between closed, half-open, and open treatment. The only recurrence occurred in a patient treated with the half-open method. Functional assessment according to the modified system recommended by Enneking demonstrated excellent functional outcomes in all patients, except one with recurrence, who was rated poor.

**Table 2:** Effect of various variables on Capanna outcome [*n* (%)]

Independent variable	Capanna grade			Overall^b^
	1 (*n* = 16)	2 (*n* = 8)	3 (*n* = 1)	(*n* = 25)
Age groups				
<11	5 (62.5)	3 (37.5)	0 (0.0)	8 (32.0)
≥11 and over	11 (64.7)	5 (29.4)	1 (5.9)	17 (68.0)
Type of cyst				
Unicameral	13 (65.0)	6 (30.0)	1 (5.0)	20 (80.0)
Aneurysmal	2 (40.0)	3 (60.0)	0 (0.0)	5 (20,0)
Activity				
Active	6 (60.0)	3 (30.0)	1 (10.0)	10 (40.0)
Latent	10 (66.7)	5 (33.3)	0 (0.0)	15 (60.0)
Craniocaudal length^a^ (cm)	7.22 ± 1.85	9.31 ± 2.75	11.50	8.06 ± 2.42
Pathologic fracture				
Yes	15 (83.3)	3 (16.7)	0 (0.0)	18 (72.0)
No	1 (14.3)	5 (71.4)	1 (14.3)	7 (28.0)
Method				
Closed	3 (100.0)	0 (0.0)	0 (0.0)	3 (12.0)
Half open	4 (44.2)	4 (44.2)	1 (11.2)	9 (36.0)
Open	9 (69.2)	4 (30.8)	0 (0.0)	13 (52.0)

^a^Means and standard deviations

^b^Column percentages are shown among the entire group

Three patients had pin-site infection, which was eliminated by orally administered antibiotic therapy, and one patient with a humeral cyst had radial-nerve neurapraxia, which spontaneously healed in 3 months.

## Discussion

First facts about distraction osteogenesis appeared in the literature in 1905 and were described by Codivilla [16], who applied the technique to elongate a femur. However, the commonly accepted “father of modern distraction osteogenesis” is G.A. Ilizarov who, in 1951, developed a technique for repairing complex fractures or nonunions of the long bones and, later, started widely applying distraction osteogenesis for treating leg-length discrepancy, nonunion, traumatic bone defects, deformity, and osteomyelitis [17]. Description of this method in managing musculoskeletal tumors has been presented in the reconstruction of diaphyseal defects after excision of tumors and for arthrodesis after joint resection [18–20].

Application of distraction osteogenesis for managing bone cysts is pathogenically defensible and, in this study, it was dictated by three main principles of treatment: (1) Destroying the fibrous wall of the cyst and, as a result, eliminating the closed cavity using corticotomy and cooptation. (2) Reducing intracystic pressure by creating an iatrogenic fracture, if absent, and inserting Kirschner wires through the cystic lesion. The role of decreased intracystic pressure was demonstrated by Shinozaki et al. [21], who treated 23 UBCs by drilling multiple holes and inserting Kirschner wires. Overall results of their study showed no recurrences, healing with residual radiolucency in eight patients, and complete healing in 15 patients. Nailing also decompresses the cyst and provides early stability to the bone, with early mobilization and fast recovery to normal activity. Elastic, stable intramedullary nailing for UBC demonstrated good to excellent results in almost all of the 47 UBCs, with long term healing within or during a 36-month period, and complications such as refracture and limb-length discrepancy [7]. In our study, we observed consolidation and cyst healing within 6 months in almost all patients. (3) Filling the pathological cystic cavity with distraction osteogenesis tissue. This step has two main advantages: it requires no auto- or allograft and simultaneously eliminates deformity and limb-length discrepancy. 

Approximately 10 % of patients in our study were at risk of growth arrest from a simple bone cyst. The reason for such arrest has not yet been determined and may be the result of a past fracture associated with the cyst, iatrogenic damage that take place as a result of surgical curettage, or of a simple bone cyst abutting the physis and disrupting the process of direct extension of the cyst through the physial plate into the epiphysis. Avoiding curettage from one side and also the possibility of managing bone length resolves the problem of limb shortening. The need for curettage is still under debate.

Some authors uniquely recommend curettage as a mandatory procedure of treatment [8, 22, 23]; others never disturb the cyst wall or its membrane [9–12, 24]. In our study, we observed no significant differences in healing time and outcome in patients treated with or without curettage. Curettage and bone grafting have been the traditional treatment of UBC. Oppenheim and Galleno [12], in 1984, reported a complication rate of 15 % in 37 patients: infection, coxa vara, epiphyseal arrest, and limb shortening. Furthermore, they found a recurrence rate of 40 %. Long periods of immobilization and difficulties obtaining adequate autograft in children are still essential parts of grafting. Besides economic aspects and availability, allograft remains a problem in some countries. Distraction osteogenesis resolves this problem.

A relatively new direction in orthopedic surgery, such as regenerative medicine, holds great promise for managing bone cysts. Instead of filling the cystic cavity with distraction osteogenesis or autograft or allograft material, trends in research revolve around regenerating damaged tissue, as well as stimulating and enhancing the body’s own repair mechanisms to heal defects and previously irreparable tissues. Great potential using osteoinductive matrix, osteoprogenitor cells, and recombinant growth factors such as bone morphogenic proteins, bone marrow, stem cells, gene therapy, and the combination of cells with biodegradable scaffolds deliver appropriate bioactive factors that may optimize this regenerative process. Krebsbach et al. [25] demonstrated in vivo bone formation after using bone-marrow-derived stem cells loaded on extracellular matrices, such as hydroxyapatite–tricalcium phosphate, applied as a scaffold. Regenerative medicine is shaping these new therapies and promising a future treatment modality for bone defects, being deemed to be and area of demand for future research, with the potential to revolutionize bone-cyst treatment.

More than half of bone cysts are first diagnosed as a pathological fracture [1]. Unlike other methods of treatment, using the method described in this study, we can begin immediate treatment without waiting of fracture healing. Steroid injection remains a widely accepted method of treatment. However, patients had a recurrence rate of 15–88 % after three injections, with limb-length discrepancy being reported in 5–15 % of patients [23, 24].

Recent studies have reported effectiveness and high healing rates with the use of autologous bone marrow and/or demineralized bone matrix percutaneous infiltration. Di Bella et al. [14] compared multiple injections of corticosteroids with a single injection of demineralized bone matrix in association with bone marrow concentrate. They demonstrated advantages of higher healing and lower failure rates. Another study [26] reported better results using a combination injection of steroids, demineralized bone matrix, and bone marrow aspirate in comparison with either injection of steroids or curettage plus bone grafting for UBCs.

Many studies dedicated to managing bone cysts with transosseous osteosynthesis are reported in the Russian Ilizarov Scientific Centre, RISC “RTO” (Kurgan, Russia), where methods such as transverse and longitudinal overlapping, substitution with cortical autografts, etc. were used to restore the cystic cavity. One of the latest studies shows good results (92.3 %) of this treatment in 46 patients with bone cysts [27].

Despite the large number of research treatment modalities, there is still no established procedure that predicts treatment success. Patient age and cystic activity affects recurrence rates. Patients >10 years experience cyst healing at a higher rate (90 %) than those <10 years (60 %), no matter what treatment regimen is utilized [28]. In our study population, all patients <10 years had results rated from good to excellent. We also had only one recurrence, which was in a patient with an active cyst.

This study has several limitations: Despite many common features of UBC and ABC, they are two distinct, nosologic entities and show some differences in clinical behavior. It would be better to evaluate them separately. However, the similar positive results inclined us to publish them together, although we strongly recommend more aggressive treatment, with curettage and open methods for ABC in comparison with UBC. The number of patients was not adequate to conduct many of the statistical analyses, as this treatment method is not widely used. To analyze the impact of various variables on the Capanna outcome, studies with a higher number of patients or meta-analyses of studies like ours could be conducted.

Despite the fact that bone cysts were discovered about 100 years ago, they remain a persistent challenge for the treating physician. Multiple treatment options have recently been presented, but no single treatment has been established as being the best, and there is still no gold standard their management. As simple bone cysts are found in long bones in 90–95 % of patients, and taking into account our achieved positive results in almost all patients in this study, we can recommend distraction osteogenesis as a treatment option. The effectiveness of this method is the low rate of recurrences during the short period of treatment (4–6 months); simultaneous elimination of limb shortening and deformities; in the presence of pathological fracture, the ability to initiate prompt treatment without waiting for the fracture to heal; and return to normal activity in a shorter period. The economic benefits are cyst cavity restoration by the patient’s own body, and avoiding allograft with its associated cost—which remains a problem in some countries.

## References

[1] Marchiori D (1999). Clinical Imaging with skeletal, chest and abdomen.

[2] Cohen J (1977). Unicameral bone cysts: a current synthesis of reported cases. Orthop Clin North Am.

[3] Chigira M, Maehara S, Arita S, Udagawa E (1983). The aetiology and treatment of simple bone cysts. J Bone Jt Surg (Br).

[4] Komiya S, Minamitani K, Sasaguri Y (1993). Simple bone cyst: treatment by trepanation and studies of bone resorptive factors in cyst fluid with a theory of its pathogenesis. Clin Orthop.

[5] Yu J, Chang S, Suratwala S (2005). Zolendronate induces apoptosis in cells from fibrocellular membrane of unicameral bone cysts (UBC). J Orthop Res.

[6] Bowen RE, Morrissy RT (2004). Recurrence of a unicameral bone cyst in the proximal part of the fibula after en bloc resection. A case report. J Bone Jt Surg Am.

[7] De Sanctis N, Andreacchio A (2006). Elastic stable intramedullary nailing is the best treatment of unicameral bone cysts of the long bones in children?. J Pediatr Orthop.

[8] Ozaki T, Hillmann A, Lindner N, Winkelmann W (1997). Cementation of primary aneurysmal bone cysts. Clin Orthop Relat Res.

[9] Brecelj J, Suhodolcan L (2007). Continuous decompression of unicameral bone cyst with cannulated screws: a comparative study. J Pediatr Orthop B.

[10] Scaglietti O, Machete PG, Batrolozzi P (1982). Final results obtained in the treatment of bone cysts with methylprednisolone acetateand a discussion of results achieved in other bone lesions. Clin Orthop.

[11] Capanna R, Dal Monte A, Gitelis S, Campanacci M (1982). The natural history of unicameral bone cyst after steroid injection. Clin Orthop.

[12] Oppenheim WL, Galleno H (1984). Operative treatment versus steroid injection in the management of unicameral bone cysts. J Pediatr Orthop.

[13] Zamzam MM, Abak AA, Bakarman KA, Al-Jassir FF, Khoshhal KI, Zamzami MM (2009). Efficacy of aspiration and autogenous bone marrow injection in the treatment of simple bone cyst. Int Orthop.

[14] Di Bella C, Dozza B, Grisoni T, Cevolani L, Donati D (2010). Injection of demineralized bone matrix with bone marrow concentrate improves healing in unicameral bone cyst. Clin Orthop Relat Res.

[15] Enneking WF (1987) Modification of the system for functional evaluation of surgical management of musculoskeletal tumours. In: Bristol-Myers/Zimmer Orthopaedic Symposium. Limb salvage in musculoskeletal oncology. Churchill Livingstone, NewYork, pp 626–639

[16] Codivilla A (1905). On the means of lengthening, in the lower limbs, the muscles and tissues which are shortened through deformity. J Bone Jt Surg (Am).

[17] Ilizarov GA (1971). Basic principles of transosseous compression and distraction osteosynthesis. Ortop Travmatol Protez.

[18] Stoffelen D, Lammens J, Fabry G (1993). Resection of a periosteal osteosarcoma and reconstruction using the Ilizarov technique of segmental ransport. J Hand Surg (Br).

[19] Said GZ, el-Sherif EK (1995). Resection-shortening-distraction for malignant bone tumours: a report of two cases. J Bone Jt Surg (Br).

[20] Canadell J, Forriol F, Cara JA (1994). Removal of metaphyseal bone tumours with preservation of the epiphysis. Physeal distraction before excision. J Bone Jt Surg (Br).

[21] Shinozaki T, Arita S, Watanabe H (1996). Simple bone cysts treated by multiple drill-holes. Acta Orthop Scand.

[22] Dormans JP, Sankar WN, Moroz L, Erol B (2005). Percutaneous intramedullary decompression, curettage, and grafting with medicalgrade calcium sulfate pellets for unicameral bone cysts in children: a new minimally invasive technique. J PediatrOrthop.

[23] Campanacci M, Capanna R, Picci P (1986). Unicameral and aneurysmal bone cysts. Clin Orthop Relat Res.

[24] Chang CH, Stanton RP, Glutting J (2002). Unicameral bone cysts treated by injection of bone marrow or methylprednisolone. J Bone Jt Surg (Br).

[25] Krebsbach PH, Kuznetsov SA, Satomura K, Emmons RV, Rowe DW, Robey PG (1997). Bone formation in vivo: comparison of osteogenesis by transplanted mouse and human marrow stromal fibroblasts. Transplantation.

[26] Sung AD, Anderson ME, Zurakowski D, Hornicek FJ, Gebhardt MC (2008). Unicameral bone cyst: a retrospective study of three surgical treatments. Clin Orthop Relat Res.

[27] Shevcov VI, Lapinin AI, Zlobin AV (2003). Rehabilitation of patients with chronic osteomielitis and bone cysts.

[28] Baig R, Eady JL (2006). Unicameral (simple) bone cysts. South Med J.

